# SCANPY: large-scale single-cell gene expression data analysis

**DOI:** 10.1186/s13059-017-1382-0

**Published:** 2018-02-06

**Authors:** F. Alexander Wolf, Philipp Angerer, Fabian J. Theis

**Affiliations:** 1 Helmholtz Zentrum München – German Research Center for Environmental Health, Institute of Computational Biology, Munich, Neuherberg Germany; 2 Department of Mathematics, Technische Universität München, Munich, Germany

**Keywords:** Single-cell transcriptomics, Machine learning, Scalability, Graph analysis, Clustering, Pseudotemporal ordering, Trajectory inference, Differential expression testing, Visualization, Bioinformatics

## Abstract

Scanpy is a scalable toolkit for analyzing single-cell gene expression data. It includes methods for preprocessing, visualization, clustering, pseudotime and trajectory inference, differential expression testing, and simulation of gene regulatory networks. Its Python-based implementation efficiently deals with data sets of more than one million cells (https://github.com/theislab/Scanpy). Along with Scanpy, we present AnnData, a generic class for handling annotated data matrices (https://github.com/theislab/anndata).

## Background

Simple integrated analysis work flows for single-cell transcriptomic data [1] have been enabled by frameworks such as SEURAT [2], MONOCLE [3], SCDE/PAGODA [4], MAST [5], CELL RANGER [6], SCATER [7], and SCRAN [8]. However, these frameworks do not scale to the increasingly available large data sets with up to and more than one million cells. Here, we present a framework that overcomes this limitation and provides similar analysis possibilities. Moreover, in contrast to the existing R-based frameworks, SCANPY’s Python-based implementation is easy to interface with advanced machine-learning packages, such as TENSORFLOW [9].

## Results

### SCANPY integrates canonical analysis methods in a scalable way

**Fig. 1:**
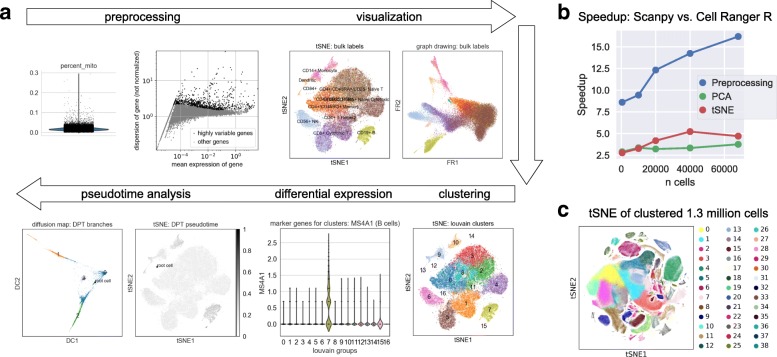
**a**SCANPY’s analysis features. We use the example of 68,579 peripheral blood mononuclear cells of [6]. We regress out confounding variables, normalize, and identify highly variable genes. TSNE and graph-drawing (Fruchterman–Reingold) visualizations show cell-type annotations obtained by comparisons with bulk expression. Cells are clustered using the Louvain algorithm. Ranking differentially expressed genes in clusters identifies the MS4A1 marker gene for B cells in cluster 7, which agrees with the bulk labels. We use pseudotemporal ordering from a root cell in the CD34+ cluster and detect a branching trajectory, visualized with TSNE and diffusion maps. **b** Speedup over CELL RANGER R kit. We consider representative steps of the analysis [6]. **c** Visualizing and clustering 1.3 million cells. The data, brain cells from E18 mice, are publicly available from 10x Genomics. PCA = principal component analysis, DC = diffusion component

SCANPY integrates the analysis possibilities of established R-based frameworks and provides them in a scalable and modular form. Specifically, SCANPY provides preprocessing comparable to SEURAT [10] and CELL RANGER [6], visualization through TSNE [11, 12], graph-drawing [13–15] and diffusion maps [11, 16, 17], clustering similar to PHENOGRAPH [18–20], identification of marker genes for clusters via differential expression tests and pseudotemporal ordering via diffusion pseudotime [21], which compares favorably [22] with MONOCLE 2 [22], and WISHBONE [23] (Fig. 1a).

### SCANPY is benchmarked in comparisons with established packages

In a detailed clustering tutorial of 2700 peripheral blood mononuclear cells (PBMCs), adapted from one of SEURAT’s tutorials (http://satijalab.org/seurat/pbmc3k_tutorial.html) [2], all steps starting from raw count data to the identification of cell types are carried out, providing speedups between 5 and 90 times in each step (https://github.com/theislab/scanpy_usage/tree/master/170505_seurat). Benchmarking against the more run-time optimized CELL RANGER R kit [6], we demonstrate a speedup of 5 to 16 times for a data set of 68,579 PBMCs (Fig. 1a,b, https://github.com/theislab/scanpy_usage/tree/master/170503_zheng17) [6]. Moreover, we demonstrate the feasibility of analyzing 1.3 million cells without subsampling in a few hours of computing time on eight cores of a small computing server (Fig. 1c, https://github.com/theislab/scanpy_usage/tree/master/170522_visualizing_one_million_cells). Thus, SCANPY provides tools with speedups that enable an analysis of data sets with more than one million cells and an interactive analysis with run times of the order of seconds for about 100,000 cells.

In addition to the mentioned standard clustering-based analyses approaches, we demonstrate the reconstruction of branching developmental processes via diffusion pseudotime [21] as in the original paper (https://github.com/theislab/scanpy_usage/tree/master/170502_haghverdi16), the simulation of single cells using literature-curated gene regulatory networks based on the ideas of [24] (https://github.com/theislab/scanpy_usage/tree/master/170430_krumsiek11), and the analysis of deep-learning results for single-cell imaging data [25] (https://github.com/theislab/scanpy_usage/tree/master/170529_images).

### SCANPY introduces efficient modular implementation choices

With SCANPY, we introduce the class ANNDATA—with a corresponding package ANNDATA—which stores a data matrix with the most general annotations possible: annotations of observations (samples, cells) and variables (features, genes), and unstructured annotations. As SCANPY is built around that class, it is easy to add new functionality to the toolkit. All statistics and machine-learning tools extract information from a data matrix, which can be added to an ANNDATA object while leaving the structure of ANNDATA unaffected. ANNDATA is similar to R’s EXPRESSIONSET [26], but supports sparse data and allows HDF5-based backing of ANNDATA objects on disk, a format independent of platform, framework, and language. This allows operating on an ANNDATA object without fully loading it into memory—the functionality is offered via ANNDATA’s backed mode as opposed to its memory mode. To simplify memory-efficient pipelines, SCANPY’s functions operate in-place by default but allow the optional non-destructive transformation of objects. Pipelines written this way can then also be run in backed mode to exploit online-learning formulations of algorithms. Almost all of SCANPY’s tools are parallelized.

SCANPY introduces a class for representing a graph of neighborhood relations among data points. The computation of neighborhood relations is much faster than in the popular reference package [27]. This is achieved by aggregating rows (observations) in a data matrix to submatrices and computing distances for each submatrix using fast parallelized matrix multiplication. Moreover, the class provides several functions to compute random-walk-based metrics that are not available in other graph software [14, 28, 29]. Typically, SCANPY’s tools reuse a once-computed, single graph representation of data and hence, avoid the use of different, potentially inconsistent, and computationally expensive representations of data.

## Conclusions

SCANPY’s scalability directly addresses the strongly increasing need for aggregating larger and larger data sets [30] across different experimental setups, for example within challenges such as the Human Cell Atlas [31]. Moreover, being implemented in a highly modular fashion, SCANPY can be easily developed further and maintained by a community. The transfer of the results obtained with different tools used within the community is simple, as SCANPY’s data storage formats and objects are language independent and cross-platform. SCANPY integrates well into the existing Python ecosystem, in which no comparable toolkit yet exists.

During the revision of this article, the loom file format (https://github.com/linnarsson-lab/loompy) was proposed for HDF5-based storage of annotated data. Within a joint effort of facilitating data exchange across different labs, ANNDATA now supports importing and exporting to loom (https://github.com/linnarsson-lab/loompy). In this context, we acknowledge the discussions with S. Linnarson, which motivated us to extend ANNDATA’s previously static to a dynamic HDF5 backing. Just before submission of this manuscript, a C++ library that provides simple interfacing of HDF5-backed matrices in R was made available as a preprint [32].

## Methods

### SCANPY’s technological foundations

SCANPY’s core relies on NUMPY [33], SCIPY [34], MATPLOTLIB [35], PANDAS [36], and H5PY [37]. Parts of the toolkit rely on SCIKIT-LEARN [27], STATSMODELS [38], SEABORN [39], NETWORKX [28], IGRAPH [14], the TSNE package of [40], and the Louvain clustering package of [41]. The ANNDATA class—available within the package ANNDATA—relies only on NUMPY, SCIPY, PANDAS, and H5PY.

SCANPY’s Python-based implementation allows easy interfacing to advanced machine-learning packages such as TENSORFLOW [9] for deep learning [42], LIMIX for linear mixed models [43], and GPY/GPFLOW for Gaussian processes [44, 45]. However, we note that the Python ecosystem comes with less possibilities for classical statistical analyses compared to R.

### Comparison with existing Python packages for single-cell analysis

Aside from the highly popular SCLVM (https://github.com/PMBio/scLVM) [46, 47], which uses Gaussian process latent variable models for inferring hidden sources of variation, there are, among others, the visualization frameworks FASTPROJECT (https://github.com/YosefLab/FastProject) [48], ACCENSE (http://www.cellaccense.com/) [49], and SPRING (https://github.com/AllonKleinLab/SPRING) [15]—the latter uses the JavaScript package (http://d3js.org D3.js for the actual visualization and Python only for preprocessing—the trajectory inference tool SCIMITAR (https://github.com/dimenwarper/scimitar), the clustering tool PHENOGRAPH (https://github.com/jacoblevine/PhenoGraph) [19], the single-cell experiment design tool MIMOSCA (https://github.com/asncd/MIMOSCA)[50], UMIS (https://github.com/vals/umis) for handling raw read data [51], the tree-inference tool ECLAIR (https://github.com/GGiecold/ECLAIR) [52], and the framework FLOTILLA (https://github.com/yeolab/flotilla), which comes with modules for simple visualization, simple clustering, and differential expression testing. Hence, only the latter provides a data analysis framework that solves more than one specific task. In contrast to SCANPY, however, FLOTILLA is neither targeted at single-cell nor at large-scale data and does not provide any graph-based methods, which are the core of SCANPY. Also, FLOTILLA is built around a complicated class STUDY, which contains data, tools, and plotting functions. SCANPY, by contrast, is built around a simple HDF5-backed class ANNDATA, which makes SCANPY both scalable and extendable (law of Demeter).

## Availability and requirements

SCANPY’s and ANNDATA’s open-source code are maintained on GITHUB (https://github.com/theislab/scanpy, https://github.com/theislab/anndata) and published under the BSD3 license.

SCANPY and ANNDATA are released via the Python packaging index: https://pypi.python.org/pypi/scanpy and https://pypi.python.org/pypi/anndata.

Demonstrations and benchmarks discussed in the main text are all stored at https://github.com/theislab/scanpy_usageand summarized here:

- Analyzing 68,579 PBMCs (Fig. 1) in a comparison with the Cell Ranger R kit [6]: https://github.com/theislab/scanpy_usage/tree/master/170503_zheng17.
- Clustering and identifying cell types, adapted from and benchmarked with http://satijalab.org/seurat/pbmc3k_tutorial.htmland one of Seurat’s tutorials [2]: https://github.com/theislab/scanpy_usage/tree/master/170505_seurat.
- Visualizing and clustering 1.3 million cells (Fig. 1c): https://github.com/theislab/scanpy_usage/tree/master/170522_visualizing_one_million_cells.
- Reconstructing branching processes via diffusion pseudotime [21]: https://github.com/theislab/scanpy_usage/tree/master/170502_haghverdi16.
- Simulating single cells using gene regulatory networks [24]: https://github.com/theislab/scanpy_usage/tree/master/170430_krumsiek11.
- Analyzing deep-learning results for single-cell images [25]: https://github.com/theislab/scanpy_usage/tree/master/170529_images.

The data sets used in demonstrations and benchmarks are three data sets from 10x Genomics.

Programming language: Python

Operating system: Linux, Mac OS and Windows
